# QuickStats

**Published:** 2014-11-21

**Authors:** FROM THE NATIONAL CENTER FOR HEALTH STATISTICS

**Figure:**
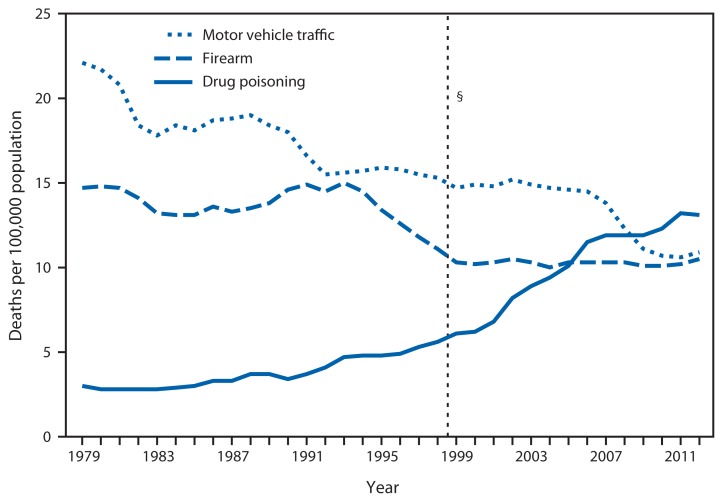
Death Rates* for Three Selected Causes of Injury^†^— National Vital Statistics System, United States, 1979–2012 * Per 100,000, age-adjusted to the 2000 U.S. standard population. ^†^ Selected because they are the most frequently occurring causes of injury deaths. Injuries are from all manners, including unintentional, suicide, homicide, undetermined intent, and legal intervention. Drug poisoning deaths include those resulting from drug overdose and other misuse of drugs. Drugs include legal and illegal drugs. ^§^ In 1999, *International Classification of Diseases, 10th Revision* (ICD-10) replaced the previous revision of the ICD (ICD-9). This resulted in approximately 5% fewer deaths being classified as motor vehicle traffic–related deaths and 2% more deaths being classified as poisoning-related deaths. Therefore, death rates for 1998 and earlier are not directly comparable with those computed after 1998. Little change was observed in the classification of firearm-related deaths from ICD-9 to ICD-10.

In 2012, a total of 41,502 drug poisoning deaths, 34,935 motor vehicle traffic deaths, and 33,563 firearm deaths occurred. The age-adjusted death rate for drug poisoning more than quadrupled from 3.0 per 100,000 in 1979 to 13.1 in 2012. In contrast, the age-adjusted rate dropped from 22.1 to 10.9 for motor vehicle traffic deaths and from 14.7 to 10.5 for firearm deaths during this period. The age-adjusted drug poisoning death rate exceeded the motor vehicle traffic death rate beginning in 2009.

**Source:** CDC WONDER, compressed mortality file, underlying cause-of-death, available at http://wonder.cdc.gov/mortsql.html.

**Reported by:** Li-Hui Chen, PhD, lchen3@cdc.gov, 301-458-4446; Andrew Fenelon.

